# Detection method for contact stress distribution of tapered roller bearings

**DOI:** 10.1038/s41598-024-61383-x

**Published:** 2024-05-07

**Authors:** Ye Ji, Xinzhong Ma, Haotian Zheng, Kun Huang, Sheng Wang, Danwen Zhang

**Affiliations:** 1 Luoyang Institute of Science and Technology, Luoyang, 471023 China; 2 Luoyang Bearing Research Institute Co., Ltd., Luoyang, 471039 China; 3 Shaanxi Business College, Xi’an, 710119 China; 4 Luoyang Juchuang Bearing Technology Co., Ltd., Luoyang, 471003 China

**Keywords:** Mechanical engineering, Software

## Abstract

The axle box of high-speed train adopts double row tapered roller bearings as transmission parts, and the reliability of bearings directly affects the safety of train operation. Tapered roller bearings can withstand axial and radial loads, their service life being closely related to the distribution of contact stress. The test object is the axle box bearing of high-speed train. According to bearing’s structural characteristics, based on the digital speckle correlation method (DSCM) and machine vision, the contact stress distribution detection devices for rollers and complete sets of bearings are developed respectively and an effective detection method is proposed. The contact stress data of the bearing which has reached the service life and the new bearing under the condition of no lubrication and grease lubrication are collected and normalized. Through the geometric relationship, based on the measured data of the selected detection points, the normal and tangential contact stress distribution of the roller and raceway under different contact conditions is obtained by data fitting. The test can be used as an evaluation basis for the effectiveness of bearing modification and provide reference for bearing design.

## Introduction

The lifespan of bearings is closely related to the magnitude and distribution of contact stress. In order to achieve a long service life, enterprises often modify the rollers and raceways when processing roller bearings. At present, the modification design and processing methods adopted by enterprises are not unique. The test is an important or even the only basis for judging the effectiveness of the modification.

As early as the 1930s, scholars began to pay attention to the ‘edge effect’ that occurs when rollers are loaded. Lundberg put forward the basic theory of generatrix modification. SKF bearing company improved the modification technology of roller bearing in 1960s^1^. Since then, scholars have analyzed and explored the ‘edge effect’ from different angles and put forward various solutions^2–7^. In China, in the 1990s, Chen et al. used the numerical solution of three-dimensional finite elastic contact problem to analyze the influence of the structural factors of the over-travel groove in the solid ring needle roller bearing on the contact pressure distribution of the logarithmic convex needle roller, and paid attention to the key factors of convex metric design^8,9^. Lv et al., used Romax Designer engineering analysis software to carry out logarithmic modification optimization analysis on the output shaft bearing of a large megawatt wind turbine gearbox^10^. Aiming at the problem that the double row tapered roller bearing for heavy truck axle had large bearing capacity and the failure mode was mostly high temperature failure, Wang et al., studied the influence of roller and raceway modification parameters on the thermal characteristics of the bearing^11^. Gong et al., aimed at the skew phenomenon of the roller during the operation of the logarithmic modified high-speed railway tapered roller bearing. Through MATLAB simulation, it was concluded that when the skew angle was small, increasing the convexity could reduce the stress concentration caused by the skew effect. When the skew angle was large, the modification method couldn’t improve the stress concentration of the roller^12^. Tian et al., analyzed the geometric characteristics of roller-whetstone contact, put forward and established the principle, method and geometric model of roller convexity modification simulation analysis, gave the simulation analysis process, and simulated and analyzed the influencing factors of convexity^13^. Xia et al., discussed the influence of roller convexity offset on the service life of tapered roller bearings, and found that the convexity center of tapered rollers could be offset to the large end of rollers in a certain range. If it was beyond this range, serious stress difference would occur at both ends of the roller, which would cause the bearing to fail in advance^14^. Due to the invisibility of the contact area, there are very few reports on experimental research on bearing contact stress, and there is a lack of systematic modification design guidance methods.

Ma et al., based on the negative Poisson’s ratio (NPR) effect obtained from the design of fiber, an auxetic structure of carbon fiber and PVA fiber was designed, then the mechanical behavior of fiber HAYs was obtained by DSCM^15^. Yu et al., proposed an enhanced full-field deformation and crack measurement method for oblique optical-axis conditions. The enhancement in the displacement measurement accuracy and the robustness of crack detection, particularly the potential of detecting hidden cracks, were verified by comparing the performance of the proposed method to that of traditional contact sensors^16^. Yang et al., developed a new experimental system in order to study the dynamic fracture behavior of rocks under an impact load with relatively low impact loading speed^17^. Zhao et al., carried out a series of three-point bending tests of granite specimens with central cracks in order to study the evolution law of the two factors during the mode I fracture evolution^18^. Li et al., used DSCM to analyze the strain field of the specimen during the whole loading process. The digital speckle strain images better proved the failure modes obtained from the experiment^19^. Most of the contact problems of mechanical parts are only theoretical analysis, such as finite element simulation, most of which lack experimental verification, and the surface profile change of contact boundary is difficult to be realized by computer simulation. Based on the existing research, DSCM is a feasible method for contact surface stress analysis of mechanical parts.

In this experiment, the DSCM is used to calculate the contact stress at the acquisition point by continuously collecting the pictures of the contact area between the roller and the raceway during the loading process, and the distribution law of the contact stress is obtained by curve fitting. The results of test and data processing can provide the basis for roller modification, and it is also the basis for measuring the effectiveness of modification design and processing.

## Experiments and methods

Two kinds of modification methods are generally used in the processing of roller bearings. One is to modify the roller only, and the other is to modify the roller and the raceway at the same time.

### Design of device for detecting only roller modification

**Figure 1:**
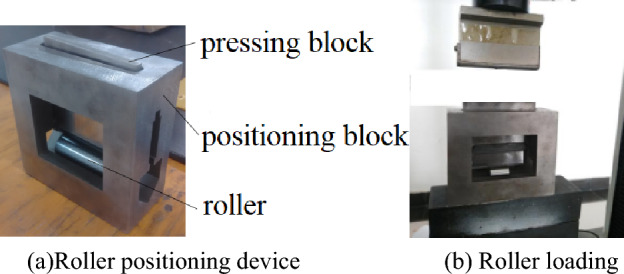
Roller contact stress detection device.

If only the roller modification is considered, the roller positioning and loading can be performed using the device shown in Fig. 1.

Figure 1a is used for roller positioning. The tensile testing machine transfers the load to the pressing block, and the pressing block is then transferred to the roller. Figure 1b shows that the roller positioning device is placed on the tensile testing machine.

**Figure 2:**
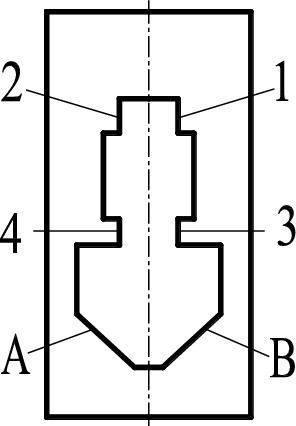
Structure of positioning block.

The Rollers are placed in a V-groove formed by two faces A and B of the positioning block. The pressing block is positioned by four contact points, expressed by 1, 2, 3 and 4. The positions of the four positioning points and the V-groove in the positioning block are shown in Fig. 2.

**Figure 3:**
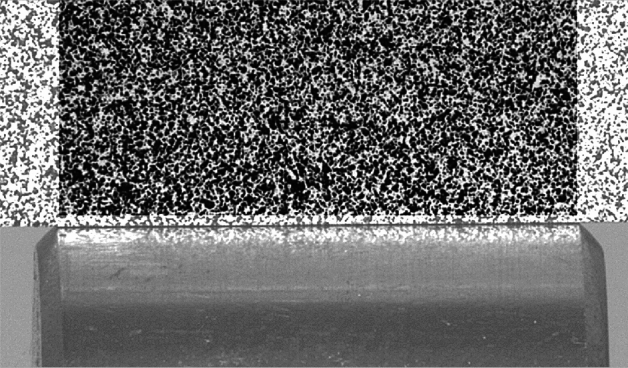
Speckle images.

The end of the pressing block is processed into a sheet of not more than 1 mm. The surface is sprayed with a layer of uniform, thin white paint, and then covered with black paint on the surface of the white paint to form dense spots. The changes of the surface spots are collected, as shown in Fig. 3.

When the DSCM system is working, the image with black spots will be continuously captured, and the surface contact stress and strain can be calculated by the change of the position of the black spots in the image. The calculation is based on pixels, and the pixel density in the horizontal and vertical directions can be set.

### Design of device for simultaneous detection of roller and raceway

**Figure 4:**
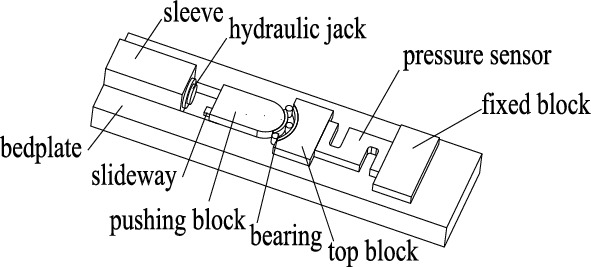
Principle diagram of loading device.

If both the roller and the raceway are modified, the contact stress distribution is closely related to the raceway shape. The detection device can be divided into three parts: loading, bearing and sensing, as shown in Fig. 4.

The device needs to consider efficiency and economy. The loading part adopts hydraulic jack, which is placed in the sleeve and can output load along the horizontal direction. The bearing part is composed of the pushing block and the cut bearing. The pushing block transmits the horizontal thrust output by the jack to the inner ring of the bearing along the radial direction. There is a top block between the pressure sensor and the bearing. One end of the sensor is placed in the groove pre-processed by the top block, and the other end is placed in the groove of the fixed block at the tail end.

The purpose of the test is to observe the deformation of the contact area between the raceway and the roller, so the bearing ring and the cage need to be cut. The outer ring is cut from the radial midline of the outer cylindrical surface by fast wire cutting, and then the ring is cut along the axis. The inner and outer rings are retained about a quarter. The wire cutting process of fast wire walking can make regular stripes appear on the surface of the ring. The stripes can be regarded as speckles, and there is no need to set speckles artificially.

**Figure 5:**
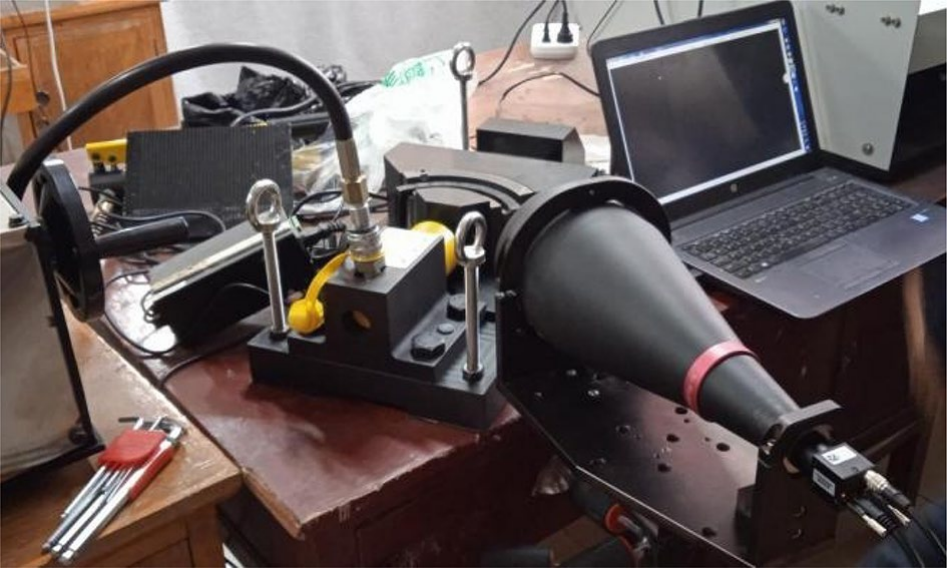
Contact stress detection system of bearing.

The mechanical device adopts a slideway to ensure that the left and right translation gaps between the push block and the top block are ± 0.05 mm, reducing or avoiding the movement of the push block and the top block. The slideway does not bear thrust, but it can meet the hydraulic jack push stroke of not less than 30 mm, and the mechanical structure is stable after loading. After several attempts and modifications of the test scheme, the developed detection system is shown in Fig. 5.

The *φ* of the detected roller is 1.5°, and *β* is 9°5′. According to the geometric relationship, it can be obtained that

1 $$\alpha \; = \;\beta + 2\varphi$$

## Analytical study

### Data acquisition

**Figure 6:**
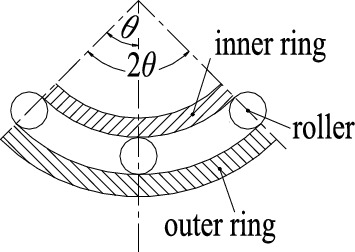
Roller distribution diagram.

Three or five rollers can be placed between a quarter of the inner and outer rings, and the system is balanced after loading. Taking three rollers as an example, the angle *θ* between two adjacent rollers is about 45°, as shown in Fig. 6.

**Figure 7:**
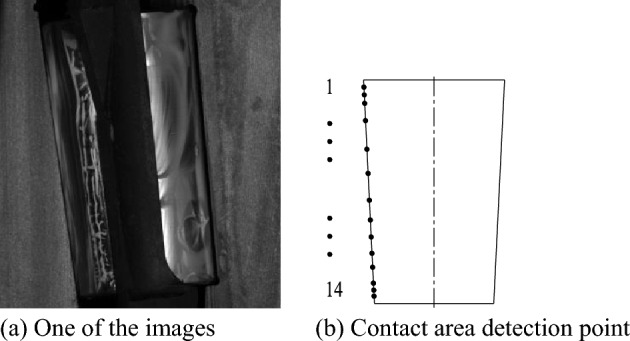
Images and detection points for calculation.

The high-definition camera is facing the bearing section to collect the strain value at the contact line between the roller and the raceway. According to the continuously changing image, as shown in Fig. 7a, DSCM can be used to calculate the horizontal and vertical stress values of the image. The section images collected by have high clarity and long processing time. Too many selected points will lead to a significant increase in calculation time, and too dense selected points will also lead to an increase in calculation time. Therefore, 14 detection points are selected on the contact line between the raceway and the roller, as shown in Fig. 7b. In order to reveal the ‘edge effect’ of the bearing roller, more detection points can be selected at both ends of the roller.

Because only the distribution law is studied, there is no need to calibrate the specific value, and the data can be normalized. The collected data are divided into two states: no lubrication and grease lubrication. The double row tapered roller bearings for high speed railway which have reached service life but can still be used and the new double row tapered roller bearings for high speed railway are tested respectively.

**Figure 8:**
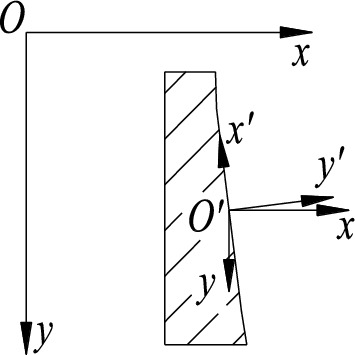
Detection coordinate system of outer ring.

The coordinate system of the visual software system is *xOy*, where the *x*-axis is positive to the right and the *y*-axis is positive to the down. The middle point of the contact line between the outer ring and the roller is *O*′, and the local coordinate system *x*′*O*′*y*′ is established. The *y*′ axis is perpendicular to the contact line, and the *x*′ axis coincides with the contact line. The direction is shown in Fig. 8, and the shadow represents the outer ring of the bearing.

### Data processing

The stress data of *x* and *y* directions in the coordinate system of the visual system are collected, and the data of each detection are normalized. The stress satisfies the Eq. (2).

2 $$\sigma_{s} = \frac{{\sigma_{i} }}{{\sigma_{\max } }}$$

The direction of *σ*_*s*_ is the same as that of *σ*_*i*_, and *σ*_*x*_ and *σ*_*y*_ can be obtained by projecting *σ*_*s*_ onto the *x*-axis and *y*-axis.

## Results and discussion

### Roller-raceway contact stress detection

Firstly, the bearing that has reached the service life is taken as the test object. After the data detected under non-lubrication conditions are processed according to Formula (2), the results projected into the vision system coordinate system are shown in Tables 1 and 2.

**Table 1:** The stress ratio *σ*_*x*_.

Detection point	Load					
	20 kg	50 kg	120 kg	200 kg	350 kg	400 kg
1	− 0.07774	− 0.13981	− 0.19967	− 0.31961	− 0.63013	− 0.76022
2	− 0.08071	− 0.13961	− 0.19544	− 0.31284	− 0.62636	− 0.75188
3	− 0.07872	− 0.11967	− 0.16752	− 0.32858	− 0.53728	− 0.64446
4	− 0.07875	− 0.13449	− 0.19568	− 0.30917	− 0.58992	− 0.66910
5	− 0.08171	− 0.11967	− 0.16752	− 0.28908	− 0.53688	− 0.64446
6	− 0.07975	− 0.13943	− 0.17941	− 0.31899	− 0.59922	− 0.74901
7	− 0.07872	− 0.11568	− 0.16194	− 0.25990	− 0.59051	− 0.61157
8	− 0.08071	− 0.11668	− 0.16362	− 0.36801	− 0.52346	− 0.78732
9	− 0.08071	− 0.11974	− 0.16334	− 0.34826	− 0.54916	− 0.76759
10	− 0.07972	− 0.11867	− 0.20173	− 0.32290	− 0.64650	− 0.77604
11	− 0.07975	− 0.11967	− 0.16660	− 0.34436	− 0.53399	− 0.63977
12	− 0.08071	− 0.13961	− 0.19544	− 0.33891	− 0.62715	− 0.81839
13	− 0.07280	− 0.15956	− 0.22337	− 0.35911	− 0.67951	− 0.82793
14	− 0.07478	− 0.16766	− 0.22364	− 0.36253	− 0.68008	− 0.83755

**Table 2:** The stress ratio *σ*_*y*_.

Detection point	Load					
	20 kg	50 kg	120 kg	200 kg	350 kg	400 kg
1	− 0.00781	− 0.01231	− 0.01797	− 0.02784	− 0.04921	− 0.05998
2	− 0.00823	− 0.01353	− 0.01904	− 0.02966	− 0.05505	− 0.06457
3	− 0.00803	− 0.01160	− 0.01632	− 0.03508	− 0.04721	− 0.05535
4	− 0.00783	− 0.01391	− 0.02073	− 0.02981	− 0.04738	− 0.05888
5	− 0.00833	− 0.01160	− 0.01632	− 0.02877	− 0.04718	− 0.05535
6	− 0.00789	− 0.01466	− 0.01799	− 0.03176	− 0.05256	− 0.06576
7	− 0.00803	− 0.01121	− 0.01577	− 0.02028	− 0.04366	− 0.03863
8	− 0.00823	− 0.01131	− 0.01414	− 0.04182	− 0.04600	− 0.07952
9	− 0.00823	− 0.01114	− 0.01592	− 0.03867	− 0.04897	− 0.07622
10	− 0.00813	− 0.01150	− 0.01965	− 0.03061	− 0.05682	− 0.06664
11	− 0.00793	− 0.01160	− 0.01322	− 0.03775	− 0.03691	− 0.05063
12	− 0.00823	− 0.01353	− 0.01904	− 0.03383	− 0.05011	− 0.07520
13	− 0.00702	− 0.01546	− 0.02176	− 0.03415	− 0.05710	− 0.07890
14	− 0.00736	− 0.01550	− 0.02006	− 0.03178	− 0.05987	− 0.08202

The relationship between the relative value of stress in the system coordinate system *xOy* and the local coordinate system* x*′*O*′*y*′ satisfies the Eqs. (3) and (4).

3 $$\sigma_{x} = - \sigma_{s} \cos \alpha - \sigma_{\tau } \sin \alpha$$

4 $$\sigma_{y} = - \sigma_{s} \sin \alpha + \sigma_{\tau } \cos \alpha$$

**Figure 9:**
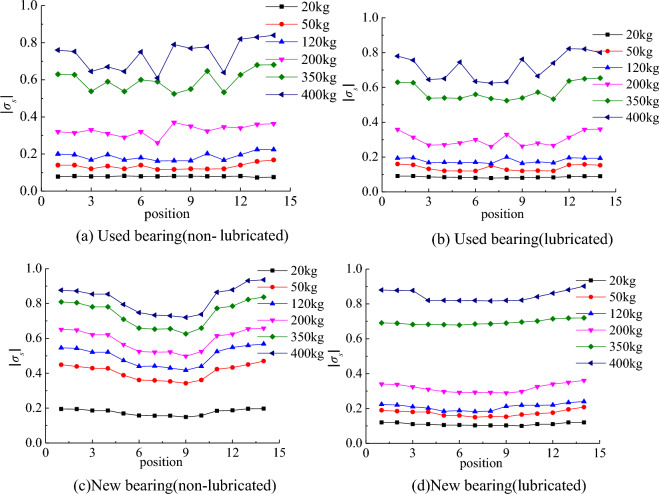
Normal contact stress distribution.

**Figure 10:**
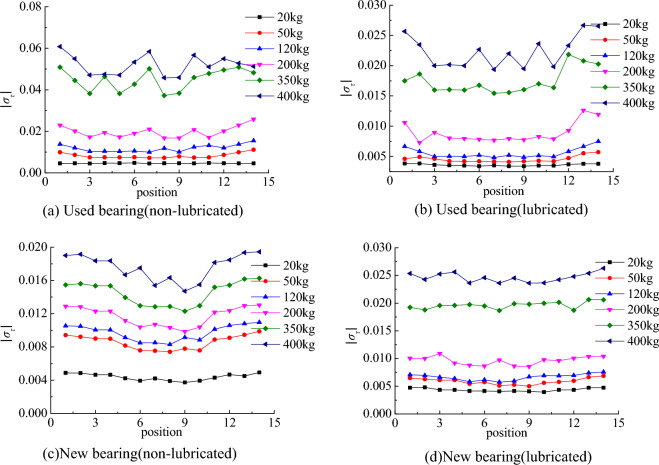
Tangential contact stress distribution.

*σ*_τ_ and *σ*_*s*_ are the relative values of tangential and normal stresses in the ring coordinate system. According to the geometric relationship, ∠*xO′y′* is equal to *α*. In order to facilitate the study of the distribution trend, both *σ*_τ_ and *σ*_*s*_ are taken as absolute values. The distribution of *σ*_*s*_ and *σ*_τ_ at 14 points on the contact line with the change of load is shown in Figs. 9a and 10a. The *σ*_*x*_ and *σ*_*y*_ of the bearings that have reached the service life in the lubricated state and the new bearings in the non-lubricated and lubricated states are no longer listed. The distribution trends of *σ*_*s*_ and *σ*_τ_ after conversion are shown in Figs. 9b–d and 10b–d, respectively.

### Bearings that have reached service life

According to the stress distribution trend obtained from the test, it can be found that there is a sudden change in the normal and tangential stress of the bearing that has reached the service life under the non-lubrication state, the regularity of the stress distribution of the roller is not obvious, and the roller has visible surface wear, which should be the reason for the irregular stress distribution. After adding grease, the variation of normal stress decreases, the irregularity of stress distribution is improved, and the tangential stress distribution is not significantly improved.

### New bearings

In the new bearing without lubrication, with the increase of load, the stress changes obviously, and the ‘edge effect’ appears. In the initial stage of increasing the radial load, the normal stress at the big end of the roller is slightly larger, and the stress at the small end increases more obviously in the later stage. After adding grease, the uniformity of normal and tangential stress distribution is obviously improved. During the test, it was found that if the grease was not evenly applied, the stress distribution would be affected, and the stress at individual points was larger.

### Regularity of distribution

Taking the new bearing detection data as the analysis object, the normal and tangential stress distribution trends are fitted by the 4th order Fourier function, the 2nd order Gaussian function and the 3rd order Sine function, respectively. The equations are shown in Eqs. (5)–(7).

5 $$S\left( n \right) = a_{0} + \sum\limits_{i = 1}^{4} {a_{i} \cos \left( {n\omega } \right)} + b_{i} \sin \left( {n\omega } \right)$$

6 $$S\left( n \right) = \sum\limits_{i = 1}^{2} {a_{i} } e^{{ - \left( {\frac{{n - b_{i} }}{{c_{i} }}} \right)^{2} }}$$

7 $$S\left( n \right) = \sum\limits_{i = 1}^{3} {a_{i} } \sin \left( {b_{i} n + c_{i} } \right)$$

where *a*_0_, *a*_*i*_, *b*_*i*_, *c*_*i*_ and *ω* are all real constants, which are determined by the relative values of the selected points and their corresponding stresses ; *n* is the selected detection point number, ranging from 1 to 14. The function value *S* (*n*) is obtained by substituting n into formula (5)–(7), and the fitting error is measured by formula (8).

8 $$F = \sqrt {\sum\limits_{n = 1}^{14} {\left[ {S\left( n \right) - \sigma_{\tau ,s} \left( n \right)} \right]^{2} } }$$

Equation (8) represents the 1-norm of a vector, and the calculation results are shown in Table 3.

**Table 3:** Fitting error.

Regularity of distribution	1-norm	Fitting function		
Normal direction (no lubrication)	*F*	4th order Fourier function	2nd order Gaussian function	3rd order Sine function
		0.0218	0.0597	0.0684
Tangential direction (no lubrication)	*F*	4th order Fourier function	2nd order Gaussian function	3rd order Sine function
		0.0064	0.0136	0.0195
Normal direction (lubrication)	*F*	4th order Fourier function	2nd order Gaussian function	3rd order Sine function
		0.0078	0.0248	0.0098
Tangential direction (lubrication)	*F*	4th order Fourier function	2nd order Gaussian function	3rd order Sine function
		0.0037	0.0065	0.0057

According to the results in Table 3, it is found that the 4th order Fourier function is closest to the detection results, indicating that the stress distribution trend of new bearings can be represented by Fourier series.

## Conclusion

Based on DSCM, a contact stress detection device for rollers and complete sets of tapered roller bearings is developed by using machine vision technology. The detection data processing method and contact stress distribution description method are proposed. When there is no lubrication and grease lubrication, the contact stress distribution of the new bearing and the bearing that has reached the service life is detected. The conclusions are as follows :

1. Roller wear will lead to uneven stress distribution of bearings. The uniform application of grease on the roller can improve the stress concentration, but it is not necessarily effective for bearings with existing surface wear.
2. The Fourier series is fitted by the selected detection point data, which can approximately represent the distribution law of bearing contact stress, and provide the basis for the modification design and evaluation of the effectiveness of the modification of tapered roller bearings.
3. The tested bearing is used in the axle box of high-speed railway train, and the new bearing has a large stress at both ends. Bearings that have reached their service life are worn, but not failed. Therefore, the edge effect can be used to evaluate the effect of bearing modification, but can not judge whether the bearing can be applied. The service life of the bearing should be determined according to the life test.

## Data Availability

The datasets generated and/or analyzed during the current study are available from the corresponding author on reasonable request.

## Abbreviations

*α*: Nominal contact angle
*φ*: Angle between roller generatrix and its center line
*β*: Angle between inner ring raceway generatrix and its center line
*σ*_*i*_: The measured stress value of a pixel point
*σ*_max_: The maximum comprehensive stress of all pixels in a single measured value
*σ*_*s*_: The dimensionless relative stress value.
*σ*_τ_: The relative values of tangential stress in the ring coordinate system.
*σ*_*s*_: The relative values of normal stress in the ring coordinate system
